# Reducing the damage of quinoa saponins on human gastric mucosal cells by a heating process

**DOI:** 10.1002/fsn3.1332

**Published:** 2019-12-06

**Authors:** Peng Xue, Lei Zhao, Yujie Wang, Zhaohua Hou, Fengxiang Zhang, Xiushi Yang

**Affiliations:** ^1^ School of Public Health and Management Weifang Medical University Weifang China; ^2^ College of Food Science and Engineering Qilu University of Technology (Shandong Academy of Sciences) Jinan China; ^3^ Institute of Crop Sciences Chinese Academy of Agricultural Sciences Beijing China

**Keywords:** GES‐1 cell, heating, liquid chromatography, quinoa saponins

## Abstract

Different food processing methods will influence the structure and activity of compounds. In this work, molecular structure and different content crude saponins that were extracted from quinoa, treated with water soaking, water boiling, and water steaming were analyzed by HPLC. Flow cytometry was employed to investigate the effects of the main saponins on the GES‐1 cell line. HPLC/MS analysis revealed that water soaking induced an extensive conversion of polar saponin Qc (424.41 ± 21.11 mg/g) to the less polar compound Qf (247.04 ± 15.71 mg/g). After treatment with 100 μg of Qf instead of Qc for 24 hr, the percentage of dead cells increased from 20.1 ± 2.2% to 86.2 ± 4.8%. One major reason of this result is that less polar saponins could damage membrane integrity more easier than polar saponins. The results indicate that saponin toxicity is enhanced after degradation, so it is necessary to avoid degradation before use.

## Graphical abstract

Thermal processing can block the hydrolysis of quinoa saponins, so reducing the damage of quinoa saponins on human gastric mucosal cells.

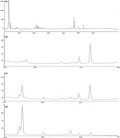

## 1 INTRODUCTION

Quinoa saponins are distributed throughout the plant, mainly in the mammary cells of the outer epidermis (bran), and the total content is approximately 2%–6% of the weight of the grain (Jarvis et al., 2017). Although saponins can protect quinoa from bird foraging, insect damage, and salt tolerance, they should be removed by grinding or washing before consumption because of their bitterness (Brito, Gosmann, & Oliveira, 2018; Gianna, Montes, Calandri, & Guzman, 2012; Gómez‐Caravaca, Iafelice, Verardo, Marconi, & Caboni, 2014; Yang et al., 2017). Previous studies were focused on the bioactive effect of saponins in quinoa, which have been proved to have anti‐inflammatory effects and to inhibit bacterial growth (Letelier, Rodríguez‐Rojas, Sánchez‐Jofré, & Aracena‐Parks, 2011; Miranda et al., 2014; Yao et al., 2015; Zhao et al., 2019).

The saponins in quinoa belong to the olean‐type saponin of pentacyclic triterpenoids, which are composed of aglycon and sugar chains, and can be divided into four groups according to the functional groups attached to C‐23 and C‐30 on the mother nucleus structure of saponins, such as oleanolic acid (OA), phytolaccagenic acid (PA), hederagenin (Hed), and serjanic acid (SA). Aglycones C‐3 and C‐28 have different amounts of structural sugar chains to replace H^+^, forming different saponins. The sugar chains linked to aglycones are mainly glucose (Glc), galactose (Gal), and arabinose (Ara), and a very few contain xylose (Xyl) and glucuronic acid (GlcUA) (Cuadrado, Ayet, & Burbano, 2012; Gil‐Ramirez et al., 2018; Li, Abliz, Tang, Fu, & Yu, 2006; Medina‐Meza, Aluwi, Saunders, & Ganjyal, 2016; Ridout, Price, Dupont, Parker, & Fenwick, 1991). Approximately 105 kinds of quinoa saponins have been detected and analyzed (Mad, Sterk, Mittelbach, & Rechberger, 2006).

It is worth noting that phytolaccagenic acid (PA) in quinoa, the main active ingredient of traditional Chinese medicine (*Phytolacca acinosa* Roxb), is a very controversial compound. PA‐type saponins from quinoa are not acutely toxic to advanced organisms (Castillo‐Ruiz et al., 2018), but PA which from *P. acinosa* Roxb could cause intestinal mucosal damage (Qi, Ma, Wang, & Zhang, 2017). Heating is a regular food processing method, and it could be optimized to have less effects on nutritional properties. For active substances, the heat processing could change the expected activity by transforming its chemical structure (Xue, Yao, Yang, Feng, & Ren, 2017). Most researches have focused on the retention of nutrients and the content of active ingredients (polyphenol/saponin) in quinoa, but there are few studies on the changes in the structure and function of saponins (Ramos Diaz et al., 2017; Gómez‐Caravaca, Segura‐Carretero, & Fernández‐Gutiérrez, 2011).

Quinoa produces a large amount of bran during pearled processing, and this by‐product comprises from 8% to 12% of the grain weight and contains from 200 to 300 g/kg saponins (Stuardo & San Martín, 2008). Therefore, quinoa bran was selected as the model to discover the changes in structure and activity of saponins during processing (Ruiz et al., 2017). In this work, the content and conversion of quinoa saponins were studied under three different processing methods (boiling, steaming, and infiltrating), which correspond to the three common methods of preparing edible quinoa, such as soup, steamed bread, and salade, respectively (López, Capparelli, & Nielsen, 2011). At the same time, the main compounds before and after conversion were also evaluated for the proliferation and toxicity of normal human gastric mucosal epithelial cells (GES‐1), which form a barrier between the body and ingested substances, such as potential nutrients and toxins. The goal of this study is to determine which method can minimize the damage of quinoa saponins on human gastric mucosal cells, improve the safety of by‐products, and provide data in support for the further utilization of saponins.

## 2 MATERIALS AND METHODS

### 2.1 Chemicals

*Chenopodium quinoa* Willd was purchased from Jingle Yilong quinoa Co., Ltd. Esculentoside A with >98% purity was purchased from the Shanghai Yuanye Biological Technology Co., Ltd. Vanillin, glacial acetic acid, perchloric acid, and dimethyl sulfoxide (DMSO) were purchased from J & K. Acetonitrile and methanol were obtained from Fisher (American). DMEM, RPMI 1640 medium, and Penicillin‐Streptomycin Solution (100×) were purchased from Fisher Scientific (China) Co., Ltd. Fetal bovine serum (FBS) was obtained from GE Healthcare Bio‐Sciences. Annexin V‐FITC/PI Apoptosis Detection Kit was purchased from Thermo Fisher Scientific (China) Co., Ltd. Cell counting kit‐8 was purchased from Dojindo (China) CO., Ltd. The other chemicals were of analytical or HPLC grade. Quinoa saponins Qb–Qf were prepared by our laboratory determined by LC/MS and NMR, purity >98%.

### 2.2 Extraction and separation of the saponins

The quinoa bran was ground into powder and passed through a 60‐mesh sieve. A total of 50 g powder was soaked in 30°C water at a ratio of 1:15. After ultrasonic extraction for 30 min, the supernatant was obtained by centrifugation for 5 min at 400 *g*. The product was extracted three times with *n*‐butanol and combined. The merged solution was recovered by rotary evaporation at 60°C and then heated and evaporated at 60°C in an evaporating dish to obtain a cold soak saponin extract (CS). In addition, 50 g of powder was suspended in cold water at a ratio of 1:15 and boiled for 30 min at 100°C. The saponin was dissolved in the hot water, and the subsequent treatment method was consistent with the method described above. Finally, the boiled quinoa saponin (BS) was obtained. Equal amounts of quinoa bran flour were wrapped with gauze and heated by steaming for 15 min at 105°C. Subsequently, the quinoa bran flour was removed and dried using a freeze dryer. The subsequent treatment method was consistent with the method described above, and steamed saponins (SS) were obtained. Then, 50 g quinoa bran flour was dispersed into 750 ml of 70% methanol and sonicated for 30 min. The merged solution was recovered by rotary evaporation at 60°C, and the subsequent treatment method was consistent with the method described to obtain saponins in the *n*‐butanol layer.

### 2.3 Preparation of saponin standards and crude quinoa saponins

Stock solutions were prepared by dissolving esculentoside A (10.1 mg) in 70% (v/v) ethanol (100 ml). A total of 12.1 mg, 10.71 mg, and 13.1 mg of crude saponins from CS, BS, and SS were dissolved in 100 ml 70% (v/v) ethanol, respectively. Quinoa saponins Qb–Qf, (1.04 mg; 1.12 mg; 1.07 mg; 1.32 mg; 1.14 mg) were dissolved in 10 ml 70% (v/v) ethanol.

### 2.4 Determination of total saponin content by spectrophotometry

The total saponins content was evaluated by using a previously reported spectrophotometry method with some modifications (Chen, Xie, & Gong, 2007). Briefly, 0, 0.2, 0.4, 0.6, 0.8, or 1.0 ml esculentoside A was added to a 10 ml glass tube and dried in a water bath at 70°C. Then, 0.2 ml vanillin‐glacial acetic acid solution and 0.8 ml perchloric acid solution were added to the 10 ml glass tube. After shaking, the tubes were heated in a constant temperature water bath at 60°C for 15 min and then cooled under flowing cold water for 5 min. Then, 5 ml glacial acetic acid was added, and the mixture was allowed to stay for 30 min. The absorbance at 560 nm was then measured by visible spectrophotometry. The absorbance value was set to a standard curve on the ordinate. Meanwhile, the absorbance value of the 0.5 ml was added to the solution in the above manner, a solution without the sample was used as a blank, and the saponins content was determined using the standard curve.

### 2.5 HPLC analyses

Chromatographic analysis was performed using a SHIMADZU Prominence LC‐20A HPLC instrument (Shimadzu Corporation) equipped with a YMC‐ODS Pack column (4.6 mm × 250 mm, YMC Co., Ltd.). The detection wavelength was set at 202 nm and the temperature of the column oven was 25°C. The mobile phase was consisted of water (A) and acetonitrile (B). A gradient elution was used as follows: 5 min 10% B; 10 min 15% B; 15 min 20% B; 35 min 28% B; 50 min 40% B; 60 min 60% B; 70 min 70% B; and 75 min 10% B. The flow rate was kept at 1 ml/min, and the injected volume was 10 μl.

### 2.6 HPLC‐ESI‐MS conditions

The column effluent of HPLC was introduced into an Agilent LC‐1100 mass spectrometer (Agilent) equipped with an electronic spray ionization source 6460 (ESI, Agilent). The parameters of the ESI were set according to a previous report with slight modifications (Gómez‐Caravaca et al., 2011). Briefly, the collision gas (N_2_) rate was maintained at 10 ml/min, and the column oven was maintained at 25°C. ESI‐MS were acquired in negative mode to generate [M−H]^−^ and ginsenoside ions by full scanning *m*/*z* over 50–2,000. The spray voltage was 4.5 kV, the capillary voltage was 10 V, and the capillary temperature was 250°C.

### 2.7 Preparation of quinoa saponins curves

The injected volume of five quinoa saponins (Qb–Qf) was 1 μl, 2 μl, 4 μl, 8 μl, 10 μl, and 12 μl, respectively. The working standard solutions were analyzed by the established method in triplicate. Calibration curves were plotted as the peak area (*y*) versus the amount of each ginsenoside standard (*x*). The content of ginsenoside in each sample was evaluated by the standard curve for each analyte. A recovery test was performed by a previously reported method (Liu et al., 2015). The spiked samples were analyzed in triplicate by the described HPLC method.

### 2.8 Cell culture

The GES‐1 cells (American Type Culture Collection, ATCC) were cultured in RM1640 growth medium containing 50 units/ml penicillin, 50 mg/ml streptomycin, and 5% FBS (Gibco Life Technologies). The cells were maintained in 5% CO_2_ in an incubator at 37°C.

### 2.9 Cell cytotoxicity assay

The quinoa saponins cytotoxicity activity was assayed as described by Yang and Liu with slight modifications (Yang & Liu, 2009). Briefly, 200 μl of GES‐1 cells were added to a 96‐well plate (5 × 10^4^ cells/well) and incubated at 37°C for 24 hr. Then, different concentrations (1, 50, 100, 200 μg/ml) of samples were mixed with the cells and incubated for 24 hr. For cytotoxicity testing, the growth medium was removed, and the cells were suspended in PBS. Next, 10 μl of WST‐8 [2‐(2‐methoxy‐4‐nitrophenyl)‐3‐(4‐nitrophenyl)‐5‐(2,4‐disulfophenyl)‐2H‐tetrazolium, monosodium salt was added to each well and incubated for approximately 1 hr. After removal of WST‐8, the 96‐well plate was completely washed by immersion in Pall water and 100 μl of elution solution (with PBS plus 50% of ethanol and 1% of acetic acid) was added. In the end, the cells were incubated for 20 min with gentle rotation at room temperature. The results were expressed in the terms of optical density at 570 nm using a Thermo Microplate Reader (Dynex Technologies).

### 2.10 Cell proliferation assay

The proliferation activity was evaluated by the method reported by You, Zhao, Liu, and Regenstein (2011). Cells were seeded (2.5 × 10^4^ cells/well) in a 96‐well flat‐bottom plate. The effect of quinoa saponins on cell viability was determined using the WST‐8 to formazan assay. After incubation at 37°C for 6 hr, the growth medium was replaced with media containing ginsenosides at variable concentrations. The absorbance of each well was read at 570 nm using a microplate reader, and the cell proliferation (percent) was determined at 96 hr from the absorbance of the sample compared to the control.

### 2.11 Apoptosis assay by flow cytometry

The GES‐1 apoptosis assay by flow cytometry followed a reported method (Cui et al., 2010). Cells were cultured as described above and treated with different concentrations of crude saponins, or saponin Qc or Qe (10, 50 and 200 μg/ml) for 24 hr, and then, the cells were rinsed with PBS and harvested in buffer (PBS–0.05% trypsin). The apoptosis of the GES‐1 cells was determined using the Annexin V‐FITC/PI apoptosis detection kit according to the manufacturer's protocol. A flow cytometer was used to analyze the fluorescence of the GES‐1 cells.

### 2.12 Data analysis

The data are presented as the mean of three replicates ± standard deviation. One‐way ANOVA with Duncan's multiple range test was used to analyze the results with SPSS 13.0 and Sigma Plot 10.0, respectively. A *p* value of <.05 was defined as statistically significant.

## 3 RESULTS AND DISCUSSION

### 3.1 Total saponins in the extracts by spectrophotometry

The regression equation of the esculentoside A standard curve was obtained as *Y* = 31.316*X *− 0.0051 (*R* ^2^ = .998) and is shown in Table 1. It showed that the linear relationship was good in 0–0.017 mg/ml. The content of the n‐butanol layer and total saponins from quinoa bran, CS, BS, and SS were also listed in Table 1. There were significant differences for the n‐butanol layer and total saponins in the methanol extract and the three different processing methods (*p* < .05).

**Table 1:** Analytical characteristics of saponins from quinoa (mg/g)

Peak	Saponins	Retention time	Calibration curve	*R* ^2^	Extract	CS	BS	SS
1	Qb	44	*Y* = 223752*X *− 126964	.99	—	90.28 ± 4.45^b^	46.36 ± 2.54^a^	92.25 ± 4.78^b^
2	Qc	46	*Y* = 890,226.64*X *− 1533636.8	.996	424.41 ± 21.11^d^	67.64 ± 2.26^a^	173.32 ± 12.43^b^	302.86 ± 19.51^c^
3	Qd	50	*Y* = 2,662,429.8*X *− 2107937.4	.993	40.18 ± 2.18^a^	—	66.59 ± 3.46^b^	61.045 ± 3.74^b^
4	Qe	53	*Y* = 343,494.34*X* + 2,448.5	.996	35.53 ± 1.87^a^	63.96 ± 3.54^c^	51.25 ± 3.78^b^	62.23 ± 3.11^c^
5	Qf	56	*Y* = 318993*X *− 60733	.998	50.39 ± 1.49^b^	247.04 ± 15.71^d^	166.29 ± 12.95^c^	35.73 ± 2.43^a^
	Total n‐butanol content				84 ± 3.6^b^	72 ± 4.48^a^	79 ± 3.11^b^	80 ± 4.61^b^
	Total saponins in n‐butanol				550.18 ± 22.31^c^	468.92 ± 20.11^a^	503.82 ± 25.32^b^	554.10 ± 24.58^b^
	Total saponins in n‐butanol by Spectrophotometer		*Y* = 31.316*X *− 0.0051	.998	641 ± 16.45^b^	533.65 ± 20.23^a^	546.88 ± 29.63^a^	578.27 ± 25.89^a^

Note

Means in a column with different letters (a‐c) are significantly different by Duncan's multiple range test at *p* < .05.

The n‐butanol layer (84 ± 3.6 mg/g) and saponins in the n‐butanol layer (641 ± 16.45 mg/g) from methanol extract are more than those from CS (72 ± 4.48 mg/g, 533.65 ± 20.23 mg/g), BS (79 ± 3.11 mg/g, 546.88 ± 29.63 mg/g), or SS (80 ± 4.61 mg/g, 578.27 ± 25.89 mg/g). The yield of the *n*‐butanol layer extract in quinoa bran was 8%, and the saponin purity was over 60%, as reported by others (Zhao et al., 2019). The values reported in this study for the saponins in quinoa seeds were within the range of the results previously published (Stuardo & San Martín, 2008). The saponin content can be determined initially by spectroscopy and further explored by HPLC.

### 3.2 Characterization of saponins

The linearity of the calibration curves of saponins Qb–Qc is shown in Table 1. The compounds showed good linearity (*R* ^2^ > .99). The average recovery of the saponins ranged from 98.6% to 102.9%, with a relative standard deviation of <3.0%.

**Figure 1:**
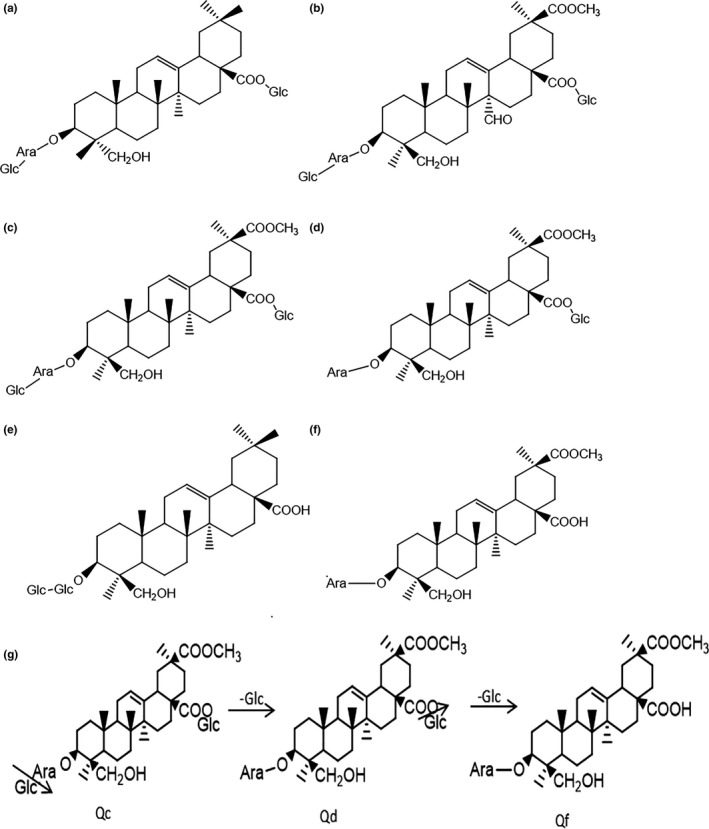
The structure of saponins from quinoa. (a) 3‐O‐β‐d‐glucopyranosyl‐(1 → 3)‐α‐l‐arabinopyranosyl hederagenin 28‐O‐β‐d‐glucopyranosyl. (b) 3‐O‐β‐d‐glucopyranosyl‐(1 → 3)‐α‐l‐arabinopyranosyl‐phytolaccagenicacid‐27‐oxo‐28‐O‐β‐d‐glucopyranosyl. (c) 3‐O‐β‐d‐glucopyranosyl‐(1 → 3)‐α‐l‐arabinopyranosyl‐phytolaccagenic acid 28‐O‐β‐d‐glucopyranosyl. (d) 3‐O‐α‐l‐arabinopyranosyl phytolaccagenic acid 28‐O‐β‐d‐glucopyranosyl ester. (e) 3‐O‐β‐d‐glucopyranosyl‐(1 → 4)‐β‐d‐glucopyranosyl‐28‐O‐hederagenin. (f) 3‐O‐α‐l‐arabinopyranosyl‐28‐O‐phytolaccagenic acid. (g) Hydrolysis processes from polar saponins Qc to Qe and Qf

**Figure 2:**
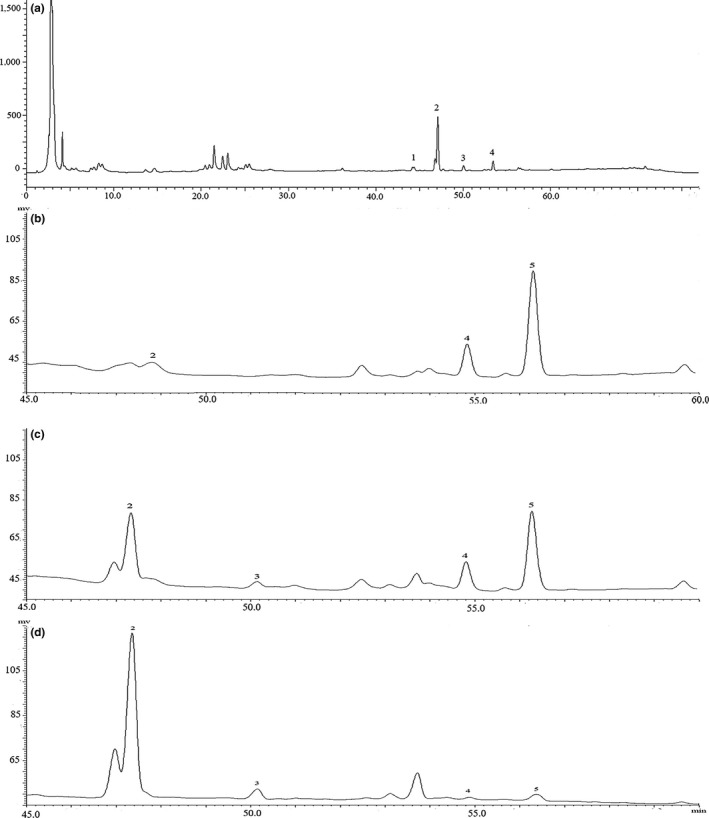
Chromatograms. Chromatogram of methanol extract (a) extraction by clod soaking (b); extraction by boiled (c); extraction by steamed (d) 80% fraction. 1, Qb; 2, Qc; 3, Qd; 4, Qe; and 5, Qf

Five reference saponins were simultaneously identified according to the standard RT (retention times) and spectra, HPLC‐ESI‐MS ion fragments, and nuclear magnetic resonance (results not shown). The structures of those saponins are summarized in Figure 1a–f. Typical HPLC‐UV chromatograms of the quinoa saponin‐enriched fractions are shown in Figure 2. The content of each saponin in every fraction is shown in Table 1. As shown in Figure 2, 5 peaks were successfully separated under the gradient elution. Consistent with the spectrophotometer results, the total saponins in the quinoa methanol extract were significantly different from the treated sample according to their origin (*p* < .05). The highest amount of total saponins in SS was 554.10 ± 24.58 mg/g, followed by those in the methanol extract (550.18 ± 22.31 mg/g), BS (503.82 ± 25.32 mg/g), and CS (468.92 ± 20.11 mg/g). A previous study reported that the husks could contain approximately 33% saponins (Stuardo & San Martín, 2008). Although most of the saponins in quinoa are polar saponins, which are soluble in water, the method for obtaining the maximum amount of saponin is the ethanol water system (Yang et al., 2017).

Quinoa bran also contained a high concentration of saponins Qb–Qf in the methanol extract, such as saponins Qc (424.41 ± 21.11 mg/g), Qd (40.18 ± 2.18 mg/g), Qe (35.53 ± 1.87 mg/g), and Qf (50.39 ± 1.49 mg/g). However, the contents of Qf that were abundantly present in CS (247.04 ± 15.71 mg/g) were significantly decreased in BS (166.29 ± 12.95 mg/g) and SS (35.73 ± 2.43 mg/g), while the contents of Qc in CS (67.64 ± 2.26 mg/g) were significantly decreased (*p* < .05) compared with those in BS (173.32 ± 12.43 mg/g) and SS (302.86 ± 19.51 mg/g).

The liquid chromatograms of the crude saponins obtained from CS, BS, and SS are shown in Figure 2. The method of water evaporation provides higher heat energy than the boiling. After different treatments, the content of saponins Qc and Qf gradually becomes significantly different (*p* < .05). With the increase in temperature, the order of the content of Qc from low to high is CS < BS < SS. Unlike the change in saponin Qc content, the content of saponin Qf gradually increases with the decrease in temperature, CS > BS > SS. This result indicates that the saponins undergo hydrolysis during the heating process, and the degree of the reaction has been directly influenced by the temperature (Brady, Ho, & Rosen, 2007).

### 3.3 Structural changes in the quinoa saponins degradation

To explore the effects of different processing on the detailed saponins profile of quinoa, these samples were also analyzed by mass spectrometry. Because the same elution conditions were used in HPLC/MS, every sample in the ion chromatograms corresponds to the UV spectra. Thus, the relative retention time and content trends for each saponin monomer are highly consistent with the UV spectra. HPLC/MS has a higher sensitivity and lower detection limit than HPLC, so more saponins are detected in the same sample. In the previous report, quinoa saponins can be divided into four categories according to the different aglycones, such as oleanolic acid (OA), phytolaccagenic acid (PA), hederagenin (Hed), and serjanic acid (SA), which are the common triterpenoid saponins (Kuljanabhagavad, Thongphasuk, Chamulitrat, & Wink, 2008). In addition to identifying the saponin species by standard compounds, the relative molecular mass and fragment ion peaks can also be utilized (Mad et al., 2006).

**Figure 3:**
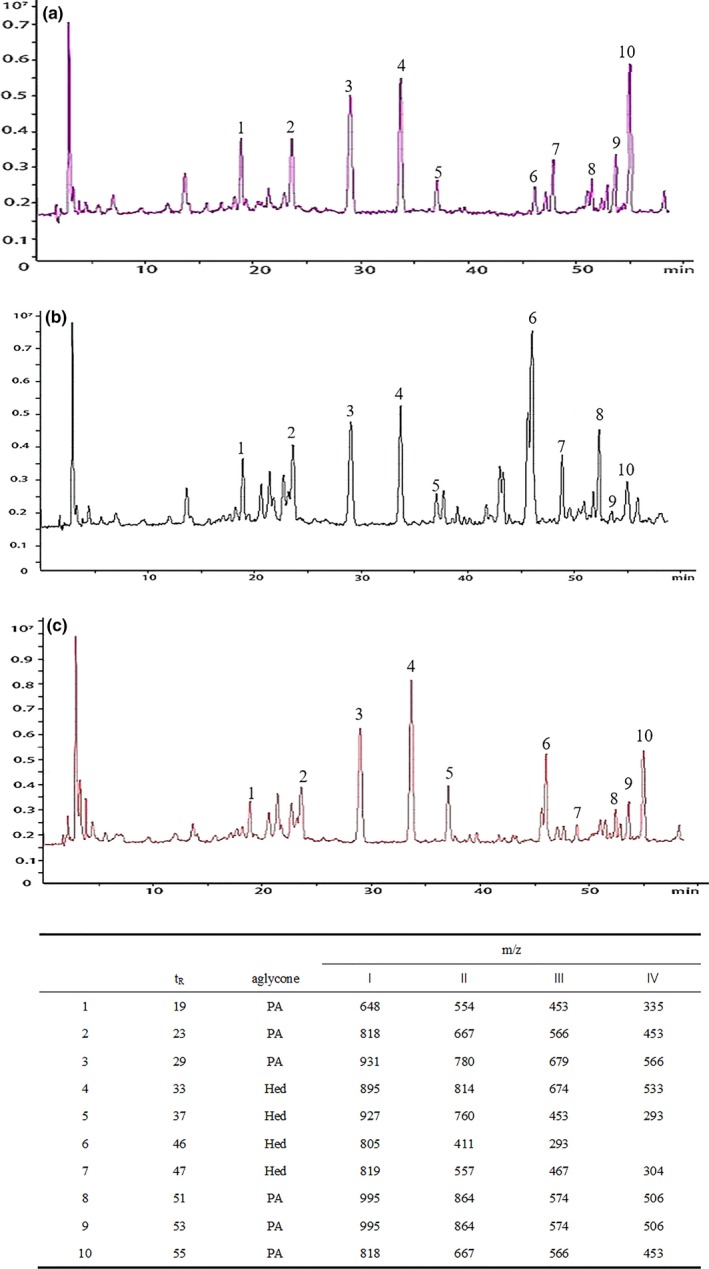
The ion flow graph of three processing methods: (a) ion flow graph of cold water immersion; (b) ion flow graph of boiled method; (c) ion flow graph of steamed method; 1. PA, 2. PA, 3. PA, 4. Hed, 5. Hed, 6. Hed, 7. Hed, 8. PA, 9. PA, and 10. PA

Saponins of peaks 1–10 labeled in the mass spectra were classified as listed in Figure 3. According to the analysis in the literature (Nickel, Spanier, Botelho, Gularte, & Helbig, 2016), using the cleavage law of ion fragments, the parent material charge‐to‐mass ratios m/z of the four aglycons are OA (*m*/*z* = 456), Hed (*m*/*z* = 472), PA (*m*/*z* = 516), and SA (*m*/*z* = 500). Many kinds of ion peak fragments may exist, such as [M+H]^+^, [M+Na]^+^, [M+2Na‐H]^+^, [M+H‐Hex]^+^, [M+H‐Pent]^+^, [M+H‐H_2_O]^+^, [M+H‐CH3]^+^, [M+H‐COOH]^+^, [M+H‐OCH_3_]^+^, etc.; OA may exist as *m*/*z* = 456, 491, 588, 654, etc.; PA may exist as *m*/*z* = 516, 648, 780, 995, etc.; and Hed may exist as *m*/*z* = 472 411, 674, 927, etc (Sun, Yang, Xue, Zhang, & Ren, 2019).

As Figure 1g shows, Qc loses one molecular glucose at the position of C‐3 and is converted to Qd, and then finally transformed to Qf. Unlike the thermal degradation of saponins in ginseng, polar ginsenosides could transform into less polar ginsenosides upon heating (Xue et al., 2017). Conversion of the quinoa saponin may be related to the enzymes present in bran, which is similar to the Oenning's study (Oenning, Juillerat, Fay, & Asp, 1994).

### 3.4 Proliferative effects of quinoa saponins on GES‐1

**Figure 4:**
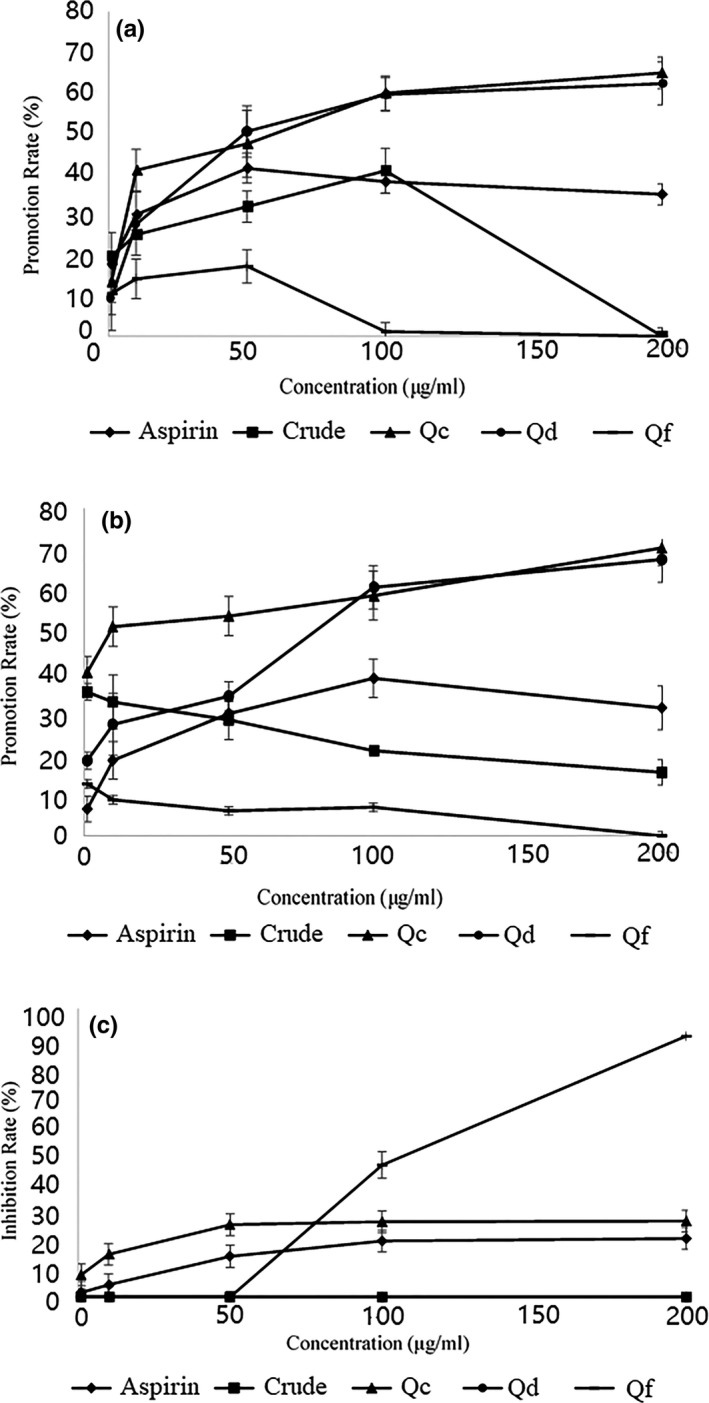
Aspirin, crude saponins, Qc, Qe, and Qf affects proliferation on GES‐1 cells. Samples improve GES‐1 cells in a concentration‐dependent manner after treatment for 12 hr (a), and 24 hr (b); samples inhibit proliferation on GES‐1 cells after treatment for 24 hr (c)

The proliferative activities of quinoa saponins toward the human gastric epithelial cell line GES‐1 at a concentration range of 1–200 μg/ml were investigated. The proliferative activity of saponin Qc and Qd toward GES‐1 cells in vitro was enhanced, as the concentration of saponin increased at 12 hr and 24 hr (Figure 4a,b). As a positive control, aspirin slightly improved GES‐1 cell proliferation at doses from 10 μg to 50 μg, but it shows a tendency to reduce the growth rate above 100 μg. The behavior of crude saponins was consistent with the positive control drug. Interestingly, saponin Qf minimally promoted cell growth.

Saponins consist of a lipophilic aglycone core and a hydrophilic sugar chain, which can perturb biologic membranes and reduce the surface tension of aqueous solutions (Bottger & Melzig, 2013). However, the activity of saponins acting on the cell membrane is mainly related to their structure (Bottger, Hofmann, & Melzig, 2012), which explains the different activities exhibited by the different saponins of quinoa.

### 3.5 Cytotoxicity effects of saponins Qc and Qf on GES‐1

The data for the cytotoxicity effects of aspirin, crude saponins and saponins Qc, Qd, and Qf on GES‐1 are shown in Figure 4c. It was observed that Qd and crude saponins did not exhibit any cytotoxicity toward GES‐1 cells. After treatment with 10 or 50 μg saponin Qf for 24 hr, inhibition of cell growth was not observed. When the dose was increased to 100 μg, the percentage of dead cells increased to 86.2 ± 4.8%. After treatment with 10, 50, 100, or 200 μg saponin Qc and aspirin for 24 hr, the percentage of dead cells increased. However, the toxicity of these saponins is much lower than saponin Qf.

**Figure 5:**
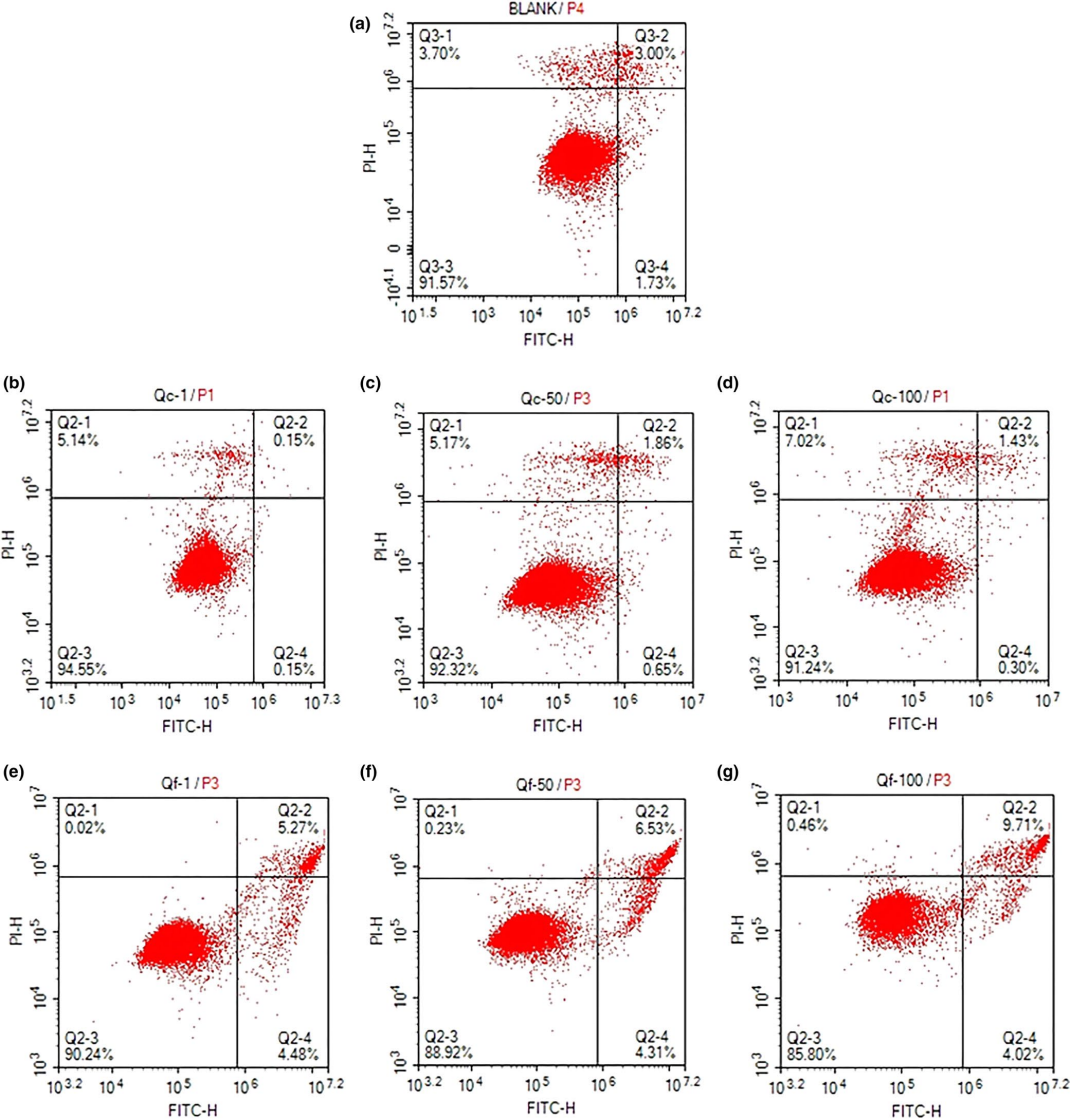
Qc and Qf induces apoptosis in GES‐1 cells. Analysis of apoptosis by staining with Annexin V‐FITC/PI. The GES‐1 cells were untreated (a) or treated with 10 (b), 50 (c), 100 μg/ml (d), Qc 10 (e), 50 (f), and 100 μg/ml (g) Qf for 24 hr. Numerical values shown in each quadrant represent the percentage of cells in that quadrant. Q3, viable cells (annexin V PI); Q1, necrotic cells (annexin V PI+); Q2, late apoptotic cells (annexin V+ PI+); Q4, early apoptotic cells (annexin V+ PI)

The apoptosis of GES‐1 cells treated with Qc and Qf was detected by flow cytometry to further verify the toxic effects of quinoa saponins. As Figure 5 shows, Qc alone at 100 μg/ml did not induce apoptosis, with 91.87% of cells remaining viable (Q3) compared to the untreated cells, with a viable cells rate of 91.57%. As shown in Figure 5e–g, after treatment with Qc (10–100 μg/ml) for 24 hr, the percentage of Q2 and Q3 was increased significantly in a concentration‐dependent manner; after treatment with 10, 50, and 100 μg/ml Qf, the total apoptotic cells increased to 9.85, 10.84, and 14.75%, respectively. The results confirmed that the ability of Qf to induce the apoptosis of GES‐1 cells was stronger than QC. Consistent with previous work, transforming ginseng by heat produced significantly higher anticancer effects than polar saponins (Quan et al., 2015). In addition, saponin anemoside B4 with five sugars had a weak cytotoxic effect on the K562, B16, Hela, and HUVEC cell lines, while pulchinenoside A with two sugars could significantly inhibit cell proliferation (Liu et al., 2015). Quinoa saponin can effectively inhibit mycelial growth and spore germination with lye treatment by destroying fungal cell membranes, which may be related to the tighter connection between saponin and cell membranes (Stuardo & San Martín, 2008). Perhaps less polar quinoa saponins are more likely to bind to cholesterol in cells alone (Bottger et al., 2012; Bottger & Melzig, 2013).

## 4 CONCLUSIONS

Most saponins in quinoa were hydrolyzed by cold soaking. One of the main reasons for this hydrolysis is that the presence of glycosidase in bran can hydrolyze glycosides from saponins because organic reagents and heating can destroy the enzyme, thereby preventing saponin hydrolysis. Quinoa saponin was more toxic to GES‐1 after hydrolysis than the polar saponin because less polar quinoa saponin is more likely to bind to cholesterol in cells. Quinoa bran contains various nutrients and could prevent endogenous enzymes from degrading saponins during consumption or feeding.

## CONFLICT OF INTERESTS

No conflict of interest was declared by the authors.

## ETHICAL APPROVAL

This study does not involve any human or animal testing.
